# Strain Optimization of Tensioned Web through Computational Fluid Dynamics in the Roll-to-Roll Drying Process

**DOI:** 10.3390/polym14122515

**Published:** 2022-06-20

**Authors:** Minho Jo, Jaehyun Noh, Gyoujin Cho, Taik-min Lee, Bukuk Oh, Sanghoon Nam, Changwoo Lee

**Affiliations:** 1 Department of Mechanical Design and Production Engineering, Konkuk University, 120 Neungdong-ro, Gwangjin-gu, Seoul 05029, Korea; als8080@konkuk.ac.kr (M.J.); zenty616@konkuk.ac.kr (J.N.); 2 Institute of Quantum Biophysics, Sungkyukwan University, 2066 Seobu-ro, Jangan-gu, Suwon 16419, Korea; gcho1004@skku.edu; 3 Korea Institute of Machinery and Materials (KIMM), Intelligence and Precision Machinery Research Division, 156 Gajeongbuk-ro, Yuseong-gu, Daejeon 34103, Korea; taikmin@kimm.re.kr; 4 Materials & Production Engineering Research Institute, LG Electronics, 222 LG-ro Jinwi-myeon, Pyeongtaek 17709, Korea; bk.oh@lge.com; 5 Department of Mechanical Engineering, Massachusetts Institute of Technology, Cambridge, MA 02139, USA; shnam@mit.edu; 6 Department of Mechanical Engineering, Konkuk University, 120 Neungdong-ro, Gwangjin-gu, Seoul 05023, Korea

**Keywords:** computational fluid dynamics (CFD), drying process, roll-to-roll (R2R), strain optimization, tensioned web, temperature distribution

## Abstract

Unpredictable web temperature distributions in the dryer and strain deviations in the cross-machine (CMD) and machine (MD) directions could hamper the manufacture of smooth functional layers on polymer-based webs through the roll-to-roll (R2R) continuous process system. However, research on this topic is limited. In this study, we developed a structural analysis model using the temperature distribution of the web as a boundary condition to analyze the drying mechanism of the dryer used in an R2R system. Based on the results of this model, we then applied structural modifications to the flow channel and hole density of the aluminum plate of the dryer. The model successfully predicted the temperature and strain distributions of the web inside the dryer in the CMD and MD by forming a tension according to the speed difference of the driven rolls at both ends of the span. Our structural improvements significantly reduced the temperature deviation of the moving web inside the dryer by up to 74% and decreased the strain deviation by up to 46%. The findings can help prevent web unevenness during the drying process of the R2R system, which is essential to minimize the formation of defects on functional layers built over polymer-based webs.

## 1. Introduction

Roll-to-roll (R2R) continuous processing has recently received increased attention in various fields as it can manufacture large-area functional layers with excellent productivity and low cost [1,2,3,4,5,6,7]. The R2R continuous process system is used to manufacture functional layers for flexible photovoltaics, electrolyte layers of solid oxide fuel cells (SOFCs), thin-film transistors (TFTs), and organic light-emitting diodes [8,9,10,11,12,13,14,15,16,17]. Many of the common problems facing various applications using R2R continuous process systems are caused by the viscoelasticity and thermal characteristics of the polymer-based web, which is the base of functional layers [18,19,20]. The web in these systems refers to the film that becomes the base of a functional layer coated or printed through slot-die coating or gravure printing. Polymer-based webs—such as polyethylene terephthalate (PET), polyimide (PI), and polyethylene naphthalate—are very thin and have excellent flexibility [21,22]. Therefore, when an operating tension is applied to the web in an R2R system, it is stretched. The web becomes more sensitive to stretching as its thickness decreases, which can seriously affect the functional layers built on it. This phenomenon causes even more serious defects when the web undergoes a high-temperature process [23,24,25].

Because the functional layer just processed on the web is often in the form of a wet liquid, the liquid must be dried through a high-temperature process. Given the nature of the R2R system, the web is also strongly influenced by the tension inside the dryer; indeed, owing to the decrease in elastic modulus with increasing drying temperature, the web inside the dryer is stretched more extensively than the web at room temperature [26,27,28]. Furthermore, the temperature at different positions of the web inside the dryer is not identical to the temperature set for the dryer, and the web forms distinct temperature distributions in the cross-machine (CMD) and machine (MD) directions in the dryer. The temperature distribution in the CMD formed on the web may cause strain deviations in this direction when an operating tension is applied to it, forming wrinkles on the web [29,30]. In addition, the thickness of the functional layer fabricated on the web may vary in the CMD according to the change principle of the contact angle of the ink due to tension [31,32,33]. The temperature distribution in the MD formed on the web causes a strain deviation in this direction; thus, the web repeatedly experiences tension in a section with a higher strain than in other sections inside the dryer and compression in a section with a lower strain, shaking up and down.

The nonuniformity of the web temperature distribution in the dryer and the resulting deviation of the strain in the CMD and MD should be resolved to enable the manufacture of functional layers on polymer-based webs through the R2R continuous process system. In recent years, many researchers have studied the high-temperature processes of the R2R system. Park et al. performed a performance analysis of the printed pattern under different drying effects according to the drying method of the R2R system (e.g., hot air, near-infrared, mid-infrared) [34]. Ham et al. conducted a study on the performance change of perovskite solar cells according to the airflow rate discharged from the dryer and solution supply rate of the slot-die coater [35]. Lee et al. proposed and verified a new control algorithm of the speed compensation scheme to alleviate tension disturbances caused by thermal deformation during the drying process [36]. However, few studies have predicted and optimized the strain distribution of the web in a high-temperature drying chamber through computational fluid dynamics (CFD) and structural analysis by uniformizing the temperature distribution of the polymer-based web formed inside the dryer in the CMD and MD based on changes in the drying mechanism inside the dryer of the R2R system.

In this study, we analyzed the drying mechanism of the dryer used in an R2R system and adjusted its flow channel and hole density such that the web inside the dryer forms a uniform temperature distribution in the CMD and MD. After a dryer with a controlled flow channel and hole density was modeled in 3D, the temperature distribution of the web inside the dryer in the CMD and MD were predicted using CFD. To predict the strain deviation due to the operating tension of the web inside the dryer, we then implemented another feature of the R2R system—that is, the tension application system—by predicting the driven roll speed difference through structural analysis. Finally, the effect of strain uniformization on dryer improvements was confirmed.

## 2. Materials and Methods

### 2.1. R2R System and Materials for the Polymer-Based Web

**Figure 1:**
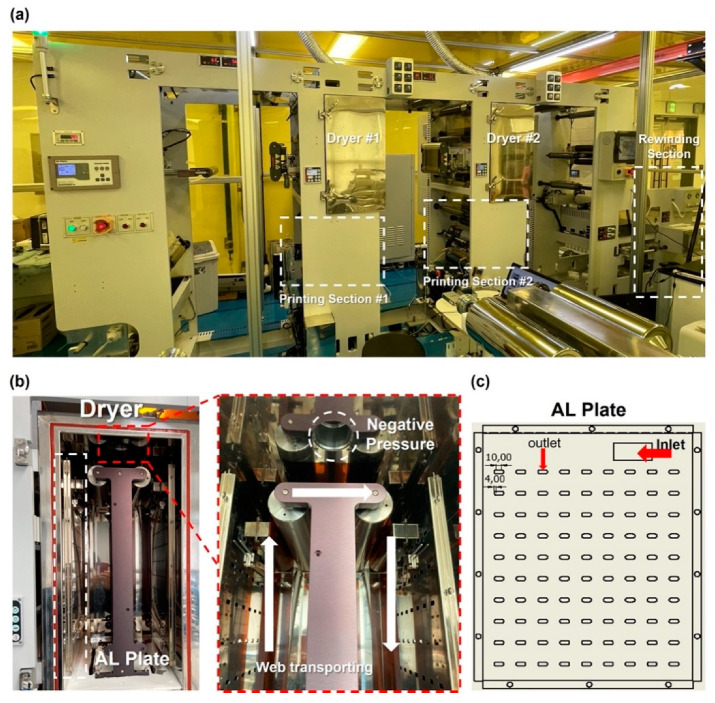
Schematics of the (**a**) R2R continuous system, (**b**) drying chamber, and (**c**) aluminum plate for hot air flow.

**Figure 2:**
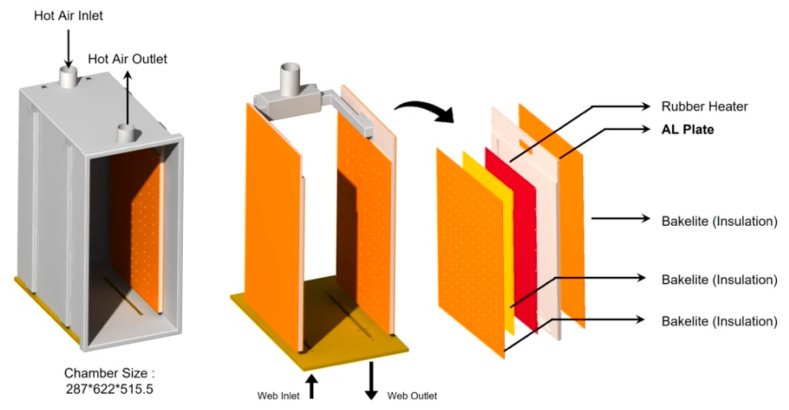
Detailed schematic of the dryer and its components.

**Figure 3:**
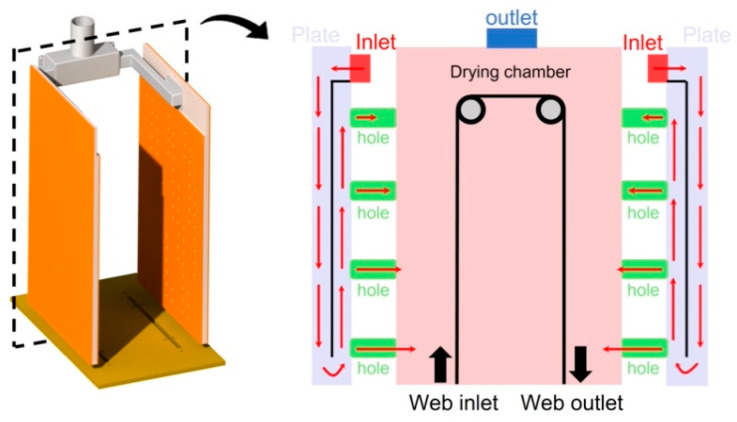
Cross-sectional view of the drying chamber and drying mechanism.

**Figure 4:**
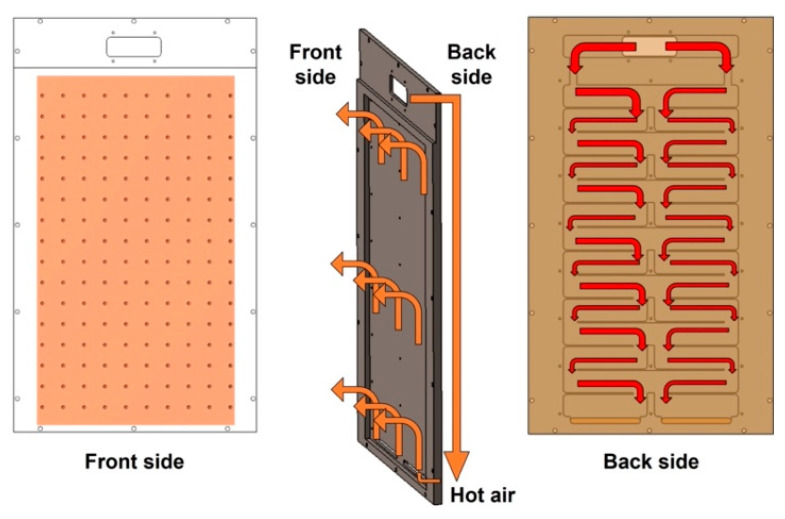
Aluminum plate for the hot air flow channel.

Figure 1 shows an R2R continuous process-based dryer used to uniformize the temperature distribution of the web inside the dryer by improving its drying mechanism. Figure 1a presents the overall schematic of the corresponding R2R system, while Figure 1b shows one of the two dryers installed in the corresponding system. Figure 1c shows the dimensions of the aluminum plate, which forms a passageway for the hot air flowing inside the dryer along its inlet and outlet. The dryer was modeled in 3D and is presented in Figure 2. Both sides of the dryer consist of three Bakelite sheets, which block heat transfer out of the dryer and provide insulating effects, a rubber heater, and an aluminum plate. Cylindrical intake and exhaust ports for supplying and discharging hot air are designed on the upper surface of the dryer. As illustrated in Figure 3, the bottom surface of the dryer has an entrance to the web, which enters vertically from the left side of the dryer, is transferred horizontally at the top, and exits vertically from the right side, as presented in the right-hand image in Figure 3. Figure 3 briefly shows the drying mechanism of the dryer in the R2R continuous process system used in this study. A hot air inlet, marked by the red square, exists on both the uppermost left and right sides of the dryer, and hot air discharged from the inlet enters the plate, marked in light purple. The plate features a partition wall, marked by a solid black line, and hot air moves from the top to the bottom of the dryer, returns to the top, and enters the drying chamber through the hole, marked by the green square. Figure 4 presents the behavior of hot air discharged from the inlet of the dryer on the plate. The initial hot air enters the backside of the aluminum plate and moves from the top to the bottom of the dryer from the left to the right to form a uniform temperature distribution on the backside of the aluminum plate (red arrow). The hot air that descends to the lowermost part of the dryer enters the front side of the aluminum plate, moves to the uppermost part once more under the exhaust pressure of the dryer, and enters the drying chamber through holes uniformly formed on the front side.

We analyzed the behavior of hot air on the backside of the aluminum plate of the drying system. Based on a process in which the hot air moves from the uppermost end to the lowermost end of the dryer in a downward zigzag pattern from side to side, the hot air collected at the center of the plate and then transferred to the front side may not be uniform in the holes because a flow channel is formed at the center of the plate at the lowermost end of the dryer. Therefore, the flow channel must be modified so that the hot air can be evenly spread at the bottom of the backside of the aluminum plate. Furthermore, on the front side of the aluminum plate, hot air moves from the lowermost end to the uppermost end of the dryer; in this case, the uniform hole distribution of the front side causes a nonuniform temperature distribution inside the dryer because a large amount of hot air is discharged from the lowermost end and a smaller amount of hot air is discharged from the uppermost end compared with that at the lowermost end. Therefore, an uneven hole distribution must be created by introducing the concept of hole density to form a uniform temperature distribution inside the dryer [37].

**Figure 5:**
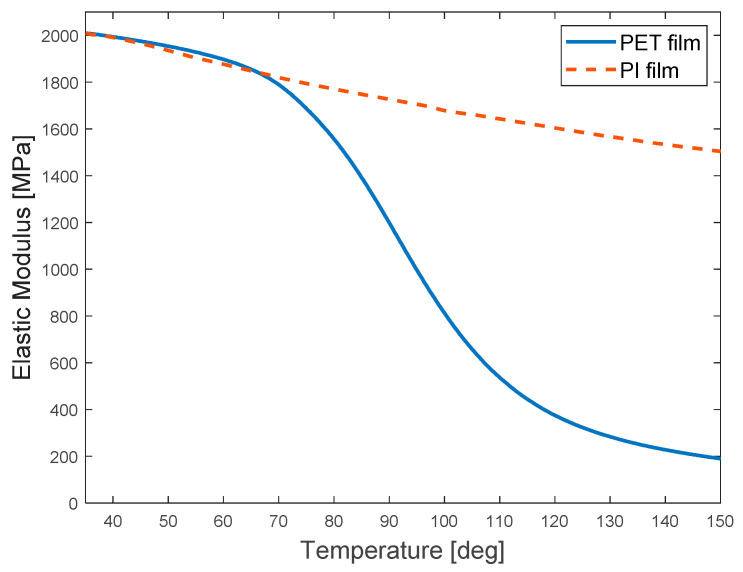
Changes in the elastic modulus of the PET and PI films as a function of temperature.

The simulation and structural analysis based on CFD were conducted using ANSYS 2022 R1 to identify the strain distribution according to the temperature distribution in the CMD and MD of the web in the current drying system. Table 1 shows the physical properties of the PET (SKC-AH71D, SKC Inc., Seoul, Korea) and PI (SKC-GF300, SKC Ink., Seoul, Korea) films used in the corresponding flow and structural analyses. PET and PI films, which are polymer-based, are mainly used in gravure printing owing to their excellent flexibility and are typically used as substrates for printed circuit boards and TFTs. They are also used to manufacture the electrolyte layers of SOFCs, which require the preparation of a large-area coating layer, such as a slot-die coating. In this simulation, changes in the elastic modulus and coefficient of thermal expansion as a function of the web temperature should be applied after the web temperature distribution inside the dryer is calculated. The change in the elastic moduli of the PET and PI films as a function of temperature is presented in Figure 5.

### 2.2. Computational Fluid Dynamics to Estimate the Temperature Distribution in the Drying Chamber

**Figure 6:**
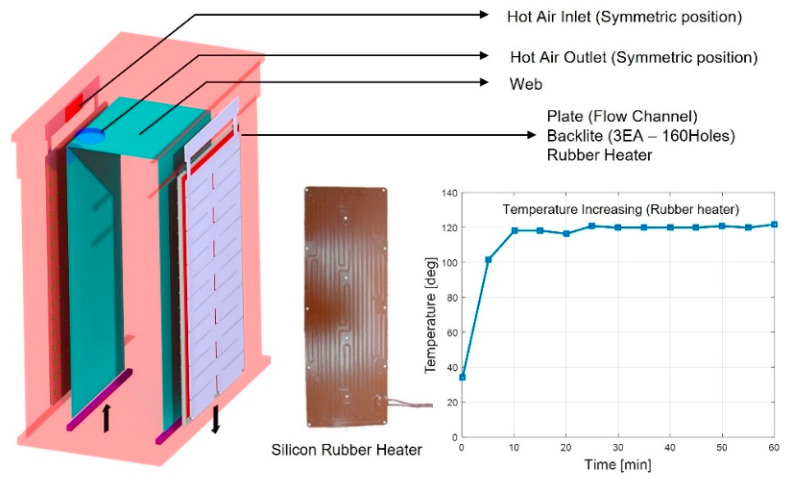
Schematic of the rubber heater and boundary conditions for the dryer simulation.

Figure 6 and Table 2 show the boundary conditions used to predict the temperature distribution inside the dryer and rubber heater, which provides an additional heat source inside the dryer. The left-hand side of Figure 6 shows the flow field inside the dryer, which is composed of a hot air inlet/outlet and rubber heater. The graph at the right-hand side of the figure shows the increase in temperature of the rubber heater over time. Because the dryer achieves a steady state after sufficient time has passed in the flow analysis simulation, the rubber heater was considered a heating element providing a temperature of 120 °C.

#### 2.2.1. Analysis of the Temperature Distribution in the Original Dryer Model

**Figure 7:**
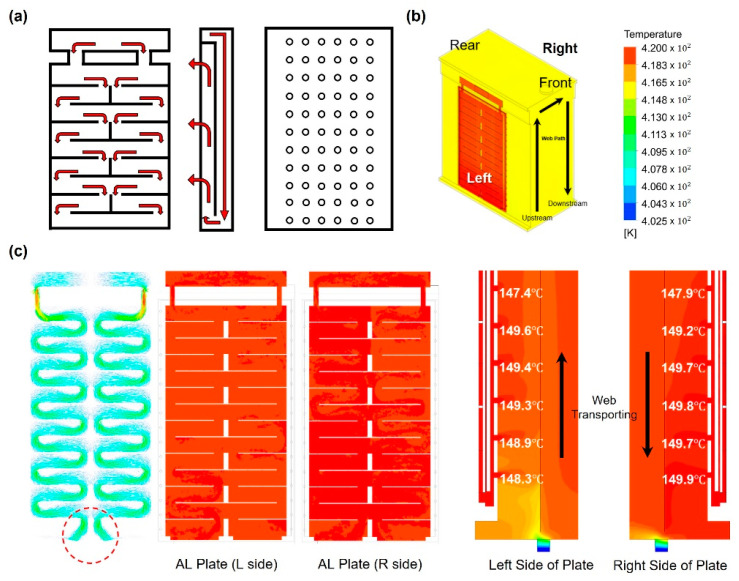
(**a**) Structure of the aluminum plate, (**b**) designation of locations for the original dryer model, and (**c**) the temperature distribution of dryer.

To investigate the nonuniformity of the web temperature distribution in the original dryer model, we established the flow channel and hole density of the aluminum plate, as shown in Figure 7a, and then performed a flow analysis. Figure 7b shows the right, left, rear, and front positions of the dryer, and Figure 7c presents the temperature analysis results for the area around the aluminum plate of the dryer. The leftmost side of Figure 7c shows the flow of hot air in the aluminum plate. The flow of high-temperature fluid in the lowermost part of the plate (red dotted circle) is concentrated at the center of the CMD. The figure on the right side also shows that the hot air flowing from top to bottom rises back to the top of the dryer and enters the dryer through the hole. Similarly, as the temperature of the hot air decreases toward the top of the dryer, the web inside the dryer may be expected to form a temperature deviation in the CMD and MD.

**Figure 8:**
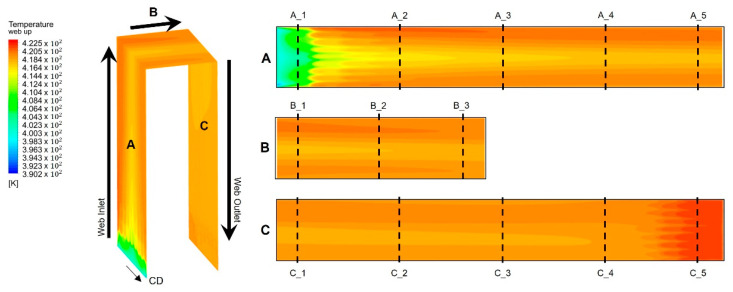
Temperature distribution of the web at Regions A, B, and C in the dryer.

Figure 8 shows the temperature distribution of the web inside the dryer according to the temperature distribution of dryer described earlier. As presented in Figure 8, three regions—A, B, and C—were defined according to the movement direction of the web inside the dryer. Region A refers to the region in which the web vertically rises after entering the dryer, region B refers to the region in which the web moves horizontally (from right to left) from the center of the dryer, and Region C refers to the region in which the web vertically descends and finally exits the dryer. Because the external low-temperature air inlet at the center of the CMD from the entrance of the dryer of Region A and the high-temperature air inlet from the aluminum plate operate simultaneously, the temperature deviation is most severe in Region A. Because Region B is located at the center of the dryer, the temperature deviation in the CMD of the web gradually decreases. Finally, in Region C, the high-temperature air at the center of the dryer is concentrated in the web movement direction, the maximum temperature is formed; thus, the temperature deviation in the CMD of the web is reduced. Table 3 shows the temperature deviation in the CMD and MD along the dotted lines (A_1, 2…) shown in the right-hand image in Figure 8. As described above, the temperature deviation in the CMD increases at the entrance of the dryer.

#### 2.2.2. Improvements in the Dryer Structure to Achieve a Uniform Temperature Distribution in the Web

**Figure 9:**
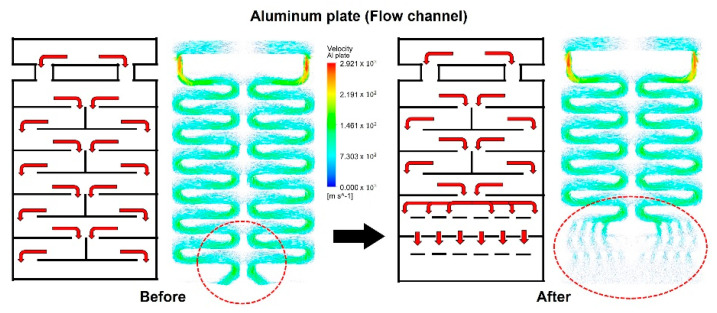
Improvements in the flow channel to achieve a uniform temperature distribution.

**Figure 10:**
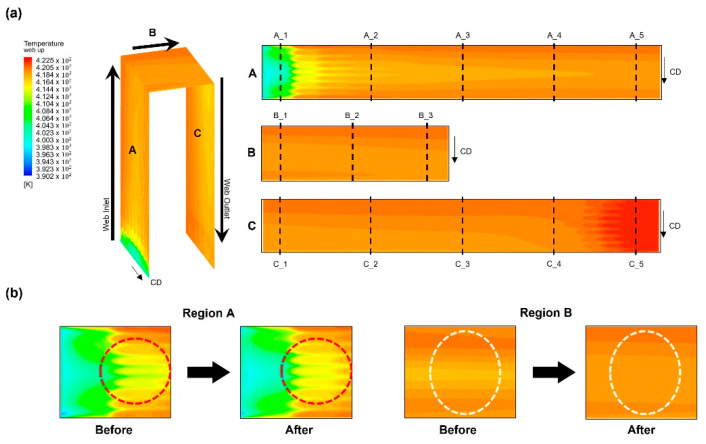
(**a**) Temperature distribution of the web in the dryer and (**b**) effect of flow channel improvements on the temperature distribution of the web.

**Figure 11:**
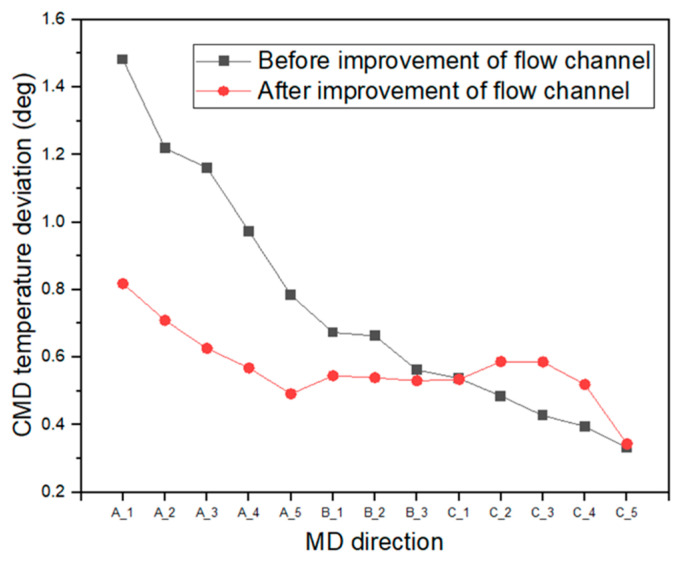
Comparison of CMD temperature deviations before and after flow channel improvements.

To uniformize the temperature distribution in the CMD and MD of the web inside the dryer, we first modified the flow channel of the aluminum plate, as presented in Figure 9. As marked by the red dotted circle in the figure, the phenomenon in which high-temperature air is concentrated at the center of the lowermost end of the plate was improved such that this air is uniformly spread to both sides of the area by modifying the flow channel. Figure 10a shows the calculation results of the temperature distribution inside the dryer after adjusting the flow channel of the aluminum plate. Figure 10b presents the difference in temperature distributions in the flow channel before and after improvement. The results indicate that the temperature deviation in the CMD of the web decreases after the improvement. In fact, as seen in Figure 11, the temperature deviation in the CMD of the dryer decreased by up to 46% in Region A after improvement. The temperature deviation in the CMD tended to increase in Region C, but was smaller than the temperature deviation at the front end of Region C, indicating that the temperature deviation in the CMD of the entire web was improved inside the dryer.

**Figure 12:**
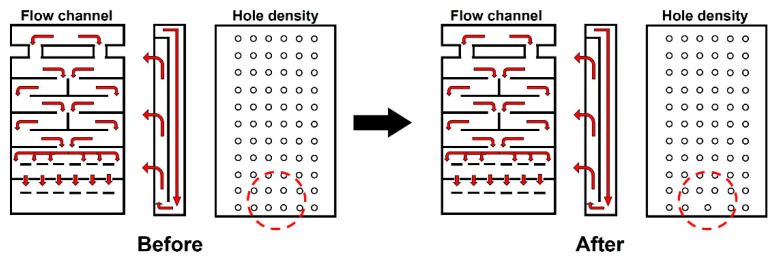
Adjustments in hole density to achieve a uniform temperature distribution.

Next, the hole density of the aluminum plate was adjusted to solve the increase in temperature deviation in the CMD of the web at the entrance of the dryer and Region C that occurs when the flow channel is changed. Assuming that the pressure of the fluid discharged from the entire holes of the aluminum plate is constant, the number of holes per unit area decreases as the distance between two holes increases; hence, the amount of hot air discharged per unit hole increases. Therefore, as shown in Figure 12, when the hole density at the lowest end of the aluminum plate is reduced, the temperature deviation decreases owing to the increase in temperature of the central part of the web at the entrance of the dryer. Similarly, the temperature deviation in Region C also decreases.

**Figure 13:**
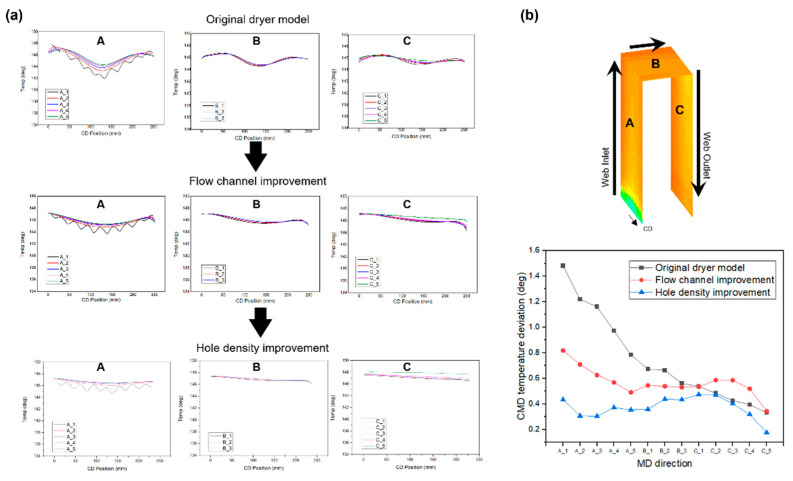
Comparison of (**a**) various dryer models and (**b**) the CMD temperature deviation at Regions A, B, and C.

A comparison of the original dryer model, flow channel of the aluminum plate, and model with adjusted hole density is illustrated in Figure 13. Figure 13a shows the temperature distributions in the CMD in Regions A, B, and C of the original dryer model, the dryer with changed flow channels, and the dryer with reduced hole density. As the curves appear to overlap in one graph, the temperature deviation in the moving direction decreases. As shown in Figure 13b, the temperature deviation of the internal web is improved by up to 74% via our two structural improvements (i.e., flow channel, hole density) in the dryer.

### 2.3. Structural Analysis to Estimate the Strain Distribution of the Moving Web in the Drying Chamber

**Figure 14:**
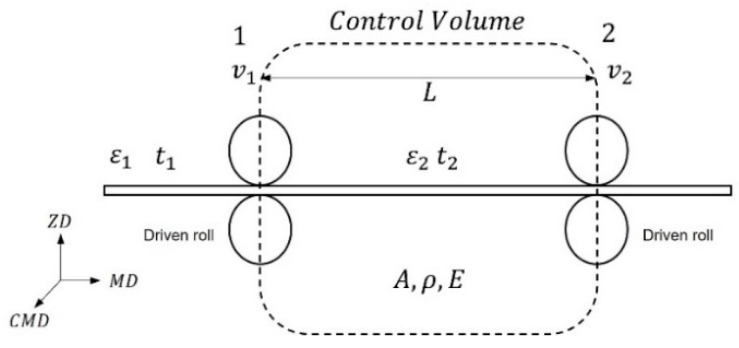
Schematic of the tension transfer mechanism.

Based on the temperature distribution of the web inside the dryer, a structural analysis was carried out to predict the strain deviation according to the temperature distribution of the web in the CMD and MD in the R2R system when an operating tension is applied to the web. As shown in Figure 14, in the R2R system, the span is defined as the gap between two driven rolls, and the tension within one span is theoretically constant. In addition, if the span is defined as a controlled volume owing to the nature of the continuous process, the amount of web entering the span and amount of web exiting the span remain constant over time according to the law of mass conservation; thus, the rate of mass change in the span is zero (under the assumption that the speed of the driven roll on the entry and exit sides does not change). For instance, if the speed of the exit driven roll increases, the amount of web exiting outside the control volume increases; thus, the tension of the web increases. In other words, the operating tension acting on the web in the R2R system is formed by the speed difference of the driven roll at the entry/exit of the span. The mechanism of tension formation in the R2R system under the influence of the speed difference of the driven roll was mathematically modeled by Shin et al., as shown in Equation (1) [38].

(1) $$\frac{d}{dt}\left\{T_{2}\left(t\right)\right\}=-\frac{v_{20}}{L}T_{2}\left(t\right)+\frac{v_{10}}{L}T_{1}\left(t\right)+\frac{AE}{L}\left\{V_{2}\left(t\right)-V_{1}\left(t\right)\right\},$$

where t,T,V,v,L,A, and E correspond to the time, tension change, speed change, initial speed, span length, web cross-sectional area, and web elastic modulus, respectively. The subscripts 1, 2, and 0 refer to the input, output, and initial values, respectively. As described above, the tension in the span is determined by the tension change $T_{1}\left(t\right)$ in the previous span and the speed difference of the entry/exit driven rolls $V_{2}\left(t\right)-V_{1}\left(t\right)$.

**Figure 15:**
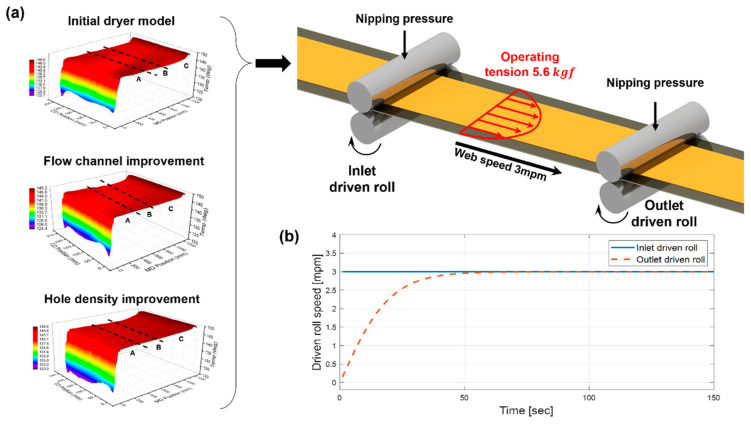
(**a**) Boundary conditions for the web strain analysis and (**b**) velocity profile of the driven roll.

To implement the tension formation mechanism by the driven roll speed difference, we set the boundary conditions as shown in Figure 15a, and then applied the web temperature distribution according to the dryer model calculated earlier to analyze the strain deviation of the web in the CMD and MD. In other words, in the structural analysis, the total strain of the web is expressed as the sum of the strains induced by thermal and elastic deformation, as shown in Equation (2).

(2) $$\varepsilon_{total}=\varepsilon_{thermal}\left(T\right)+\varepsilon_{elastic}\left(E\left(T\right)\right),$$

where ε, T, and E represent the strain, temperature, and elastic modulus, respectively. The strain caused by elastic deformation $\varepsilon_{elastic}$ is affected by the elastic modulus, which varies depending on the temperature. Figure 15b shows the speed change of the entry/exit driven rolls over time. The driven roll at the span entry side does not change its speed over time, whereas the driven roll on the exit side converges at the same speed as that at the inlet side after accelerating for a certain period of time. During the acceleration phase of the outlet driven roll, an operating tension is formed on the web, and the tension formed from the region in which the speed of the entry/exit driven rolls becomes the same remains constant. The velocity profile of the exit driven roll was applied so that the tension applied to the web was approximately 5.6 kgf.

## 3. Results and Discussion

**Figure 16:**
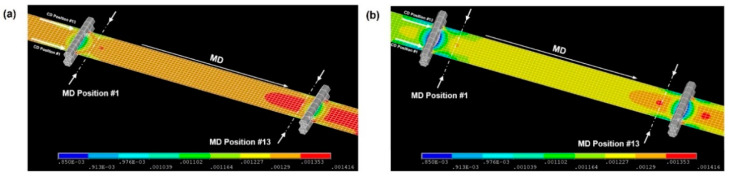
Strain distributions of the (**a**) PET and (**b**) PI films.

The strain distribution formed on the web was analyzed for the case in which the operating tension induced by the driven roll speed difference in the R2R system was applied to the web; here, the temperature distributions of the web inside the original dryer, the dryer with improved flow channels, and the dryer with improved hole density of the aluminum plate were set as boundary conditions. Figure 16 presents the strain distributions of the web inside the original dryer in the CMD and MD. Figure 16a,b show the case where the webs transferred inside the original dryer are PET and PI films, respectively. The PET film shows a greater strain because its elastic modulus is more sensitive to temperature than that of the PI film. The web in Figure 16 is divided into 13 regions in the movement direction; MD positions #1–#5 correspond to Region A, MD positions #6–#8 correspond to Region B, and MD positions #9–#13 correspond to Region C.

**Figure 17:**
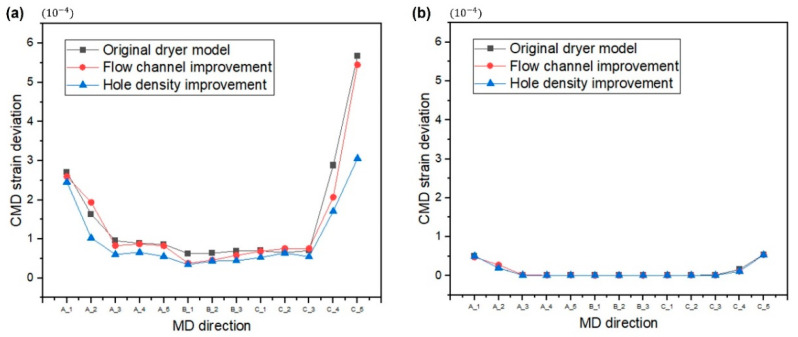
Strain distributions of the (**a**) PET and (**b**) PI films.

The same structural analysis was applied to the webs inside the dryer with improved flow channels and the dryer with improved hole density of the aluminum plate. The strain distributions are tabulated in Table 4 and compared in Figure 17. In the case of the PET film, as the temperature distribution of the web inside the dryer became more uniform, the strain deviation decreased under an operating tension. As the PI film has a much smaller strain compared with the PET film, even if the temperature distribution of the web inside the dryer was uniformized, no significant difference in strain deviation was observed. Wrinkles that may occur on the web can be prevented by reducing the strain deviation of the web inside the dryer. Wrinkles, a type of web unevenness, affect the rheology of the ink that will later be stacked or printed on the web through a coating or printing process, leading to defects (e.g., ink widening or agglomeration) [39,40]. The temperature distribution of the web inside the dryer is significantly influenced by the structure and drying mechanism of the dryer. Thus, minimizing not only the temperature deviation of the web inside the dryer—which may occur as a result of the structure and drying mechanism of the dryer—but also the strain deviation when tension is applied to the web is important.

## 4. Conclusions

In this study, we predicted the temperature distribution of a web inside the dryer according to the structure and drying mechanism of the dryer in the drying process of an R2R system. Moreover, a structural analysis model was developed by setting the temperature distribution of the web as a boundary condition. This model could predict the strain distribution of the web inside the dryer in the CMD and MD by forming a tension based on the speed difference of the driven rolls at both ends of the span. The flow channel and hole density of the aluminum plate were improved after calculating the temperature distribution of the web in the original dryer model to solve the problem of increasing temperature deviation at the entry/exit of the dryer. According to the results, the temperature deviation of the moving web inside the dryer was significantly reduced by up to 74%. A tension formation model based on driven roll speed differences was used to compare the strain distributions applied to the web in the dryer before and after the improvement. The strain deviation decreased by up to 46% in the hole density improvement model compared with that in the original dryer model. Preventing web unevenness during the drying process of the R2R system is very important to minimize possible defects, such as wrinkles or baggy webs. In particular, given the nature of the drying process, the web is located in a high-temperature environment and, thus, vulnerable to stretching under an operating tension. In this case, if the strain induced by a high temperature applied to the web is uniform in the CMD and MD under the assumption that the stress applied to the web is an elastic deformation below the yield stress, the defect does not occur. However, owing to the structure of the dryer, implementing a uniform temperature distribution of the web in the CMD and MD is very difficult. Therefore, the best approach to minimize this defect is to control the structure or drying mechanism of the dryer so that the temperature distribution of the web is formed as uniformly as possible and a uniform strain distribution is induced. Furthermore, predicting the strain distribution of webs using simulations, such as flow and structural analyses, to confirm the effect of the drying mechanism or structure of the changed dryer is very efficient in terms of cost because it saves the trouble of actually building the dryer as an improvement plan. In this case, calculating the strain distribution of the web based on the speed difference between the driven rolls, which is clearly different from that observed by stretching the web, by exerting a force on both ends of the web is important. In future work, we aim to predict the strain distribution of the web using the developed model and to prevent problems that may occur in various processes by expanding and applying the model to consider the fields of lateral dynamics, register control, and winding process.

## Figures and Tables

**Table 1:** Material properties of the PET and PI films.

Material	Properties	Unit	Value
PET (polyethylene terephthalate) film	Thickness	mm	0.10
	Width	mm	0.25
	Elastic modulus (Room temperature)	GPa	2.01
	Density	$\mathrm{kg}/m^{3}$	1450
	Thermal conductivity	W/m·K	0.290
	Coefficient of thermal expansion	/K	0.00008
PI (polyimide) film	Thickness	mm	0.10
	Width	mm	0.25
	Elastic modulus (Room temperature)	GPa	2.01
	Density	$\mathrm{kg}/m^{3}$	1420
	Thermal conductivity	W/m·K	0.120
	Coefficient of thermal expansion	/K	0.00002

**Table 2:** Specifications of the rubber heater and boundary conditions for the dryer simulation.

Rubber Heater		Boundary Conditions	
Thickness	under 1.5 mm	Hot air inlet speed	0.00598 m/s
Service voltage	AC 110 V , 220 V 60 Hz	Hot air inlet temperature	150 °C
Maximum temperature	250 °C	Hot air outlet pressure	Negative pressure
Continuous temperature limit	200 °C	Wall	No-slip condition
Insulation	Silicon rubber 0.7 mm	Rubber heater temperature	120 °C
Heat source	Ni−Cr wire	Web speed	3 mpm

**Table 3:** Standard deviations of the web temperature distribution in the dryer.

Region	Avg. Temperature	Maximum Temperature		Standard Deviation (Plane)	
A	144.2 °C	147.8 °C		3.870 °C	
B	145.7 °C	146.9 °C		0.684 °C	
C	146.1 °C	148.5 °C		0.879 °C	
Standard Deviation (Line)	1	2	3	4	5
A	1.481 °C	1.219 °C	1.161 °C	0.973 °C	0.784 °C
B	0.673 °C	0.663 °C	0.562 °C	-	-
C	0.537 °C	0.485 °C	0.427 °C	0.395 °C	0.331 °C

**Table 4:** Standard deviations of the strain distributions of the PET and PI films in the dryer.

**PET Film**																	
**Original Dryer Model**						**Flow Channel Improvement**						**Hole Density Improvement**					
**MD**	**Std.** **Deviation** **[10^−4^]**	**MD**	**Std.** **Deviation** **[10^−4^]**	**MD**	**Std.** **Deviation** **[10^−4^]**	**MD**	**Std.** **Deviation** **[10^−4^]**	**MD**	**Std.** **Deviation** **[10^−4^]**	**MD**	**Std.** **Deviation** **[10^−4^]**	**MD**	**Std.** **Deviation** **[10^−4^]**	**MD**	**Std.** **Deviation** **[10^−4^]**	**MD**	**Std.** **Deviation** **[10^−4^]**
A_1	2.7043	B_1	0.6319	C_1	0.7027	A_1	2.5953	B_1	0.3837	C_1	0.6851	A_1	2.4570	B_1	0.3481	C_1	0.5287
A_2	1.6362	B_2	0.6372	C_2	0.6508	A_2	1.9366	B_2	0.4606	C_2	0.7586	A_2	1.0269	B_2	0.4377	C_2	0.6433
A_3	0.9578	B_3	0.6971	C_3	0.7066	A_3	0.8316	B_3	0.5827	C_3	0.7559	A_3	0.6078	B_3	0.4467	C_3	0.5480
A_4	0.8910			C_4	2.8824	A_4	0.8712			C_4	2.0682	A_4	0.6607			C_4	1.7115
A_5	0.8641			C_5	5.6789	A_5	0.8279			C_5	5.4529	A_5	0.5581			C_5	3.0596
**PI Film**																	
**Original Dryer Model**						**Flow Channel Improvement**						**Hole Density Improvement**					
**MD**	**Std.** **Deviation** **[10^−4^]**	**MD**	**Std.** **Deviation** **[10^−4^]**	**MD**	**Std.** **Deviation** **[10^−4^]**	**MD**	**Std.** **Deviation** **[10^−4^]**	**MD**	**Std.** **Deviation** **[10^−4^]**	**MD**	**Std.** **Deviation** **[10^−4^]**	**MD**	**Std.** **Deviation** **[10^−4^]**	**MD**	**Std.** **Deviation** **[10^−4^]**	**MD**	**Std.** **Deviation** **[10^−4^]**
A_1	0.5092	B_1	0.0088	C_1	0.0098	A_1	0.4761	B_1	0.0043	C_1	0.0092	A_1	0.5046	B_1	0.0047	C_1	0.0071
A_2	0.1936	B_2	0.0086	C_2	0.0093	A_2	0.2770	B_2	0.0073	C_2	0.0115	A_2	0.1992	B_2	0.0058	C_2	0.0085
A_3	0.0174	B_3	0.0087	C_3	0.0200	A_3	0.0259	B_3	0.0079	C_3	0.0119	A_3	0.0157	B_3	0.0061	C_3	0.0129
A_4	0.0093			C_4	0.1616	A_4	0.0116			C_4	0.1161	A_4	0.0087			C_4	0.1137
A_5	0.0089			C_5	0.5453	A_5	0.0137			C_5	0.5362	A_5	0.0113			C_5	0.5354

## Data Availability

Data presented in this study is available upon request from the corresponding author.
